# Transcriptional analysis of the cell division-related *ssg* genes in *Streptomyce*s *coelicolor* reveals direct control of *ssgR* by AtrA

**DOI:** 10.1007/s10482-015-0479-2

**Published:** 2015-05-23

**Authors:** Songhee H. Kim, Bjørn A. Traag, Ayad H. Hasan, Kenneth J. McDowall, Byung-Gee Kim, Gilles P. van Wezel

**Affiliations:** School of Chemical and Biological Engineering and Institute of Molecular Biology and Genetics, Seoul National University, Kwanak-gu, Seoul, 151-744 Korea; Bayer CropScience LP, Biologics, 890 Embarcadero Drive, West Sacramento, CA 95605 USA; Astbury Centre for Structural Molecular Biology, Faculty of Biological Sciences, University of Leeds, Leeds, LS2 9JT UK; Molecular Biotechnology, Institute of Biology, Leiden University, PO Box 9505, 2300RA Leiden, The Netherlands

**Keywords:** SALP-family regulator, Actinomycetes, Sporulation, FtsZ, Cell cycle control

## Abstract

SsgA-like proteins are a family of actinomycete-specific regulatory proteins that control cell division and spore maturation in streptomycetes. SsgA and SsgB together activate sporulation-specific cell division by controlling the localization of FtsZ. Here we report the identification of novel regulators that control the transcription of the *ssgA*-like genes. Transcriptional regulators controlling *ssg* gene expression were identified using a DNA-affinity capture assay. Supporting transcriptional and DNA binding studies showed that the *ssgA* activator gene *ssgR* is controlled by the TetR-family regulator AtrA, while the γ-butyrolactone-responsive AdpA (SCO2792) and SlbR (SCO0608) and the metabolic regulator Rok7B7 (SCO6008) were identified as candidate regulators for the cell division genes *ssgA, ssgB* and *ssgG*. Transcription of the cell division gene *ssgB* depended on the sporulation genes *whiA* and *whiH*, while *ssgR, ssgA* and *ssgD* were transcribed independently of the *whi* genes. Our work sheds new light on the mechanisms by which sporulation-specific cell division is controlled in *Streptomyces*.

**Electronic supplementary material:**

The online version of this article (doi:10.1007/s10482-015-0479-2) contains supplementary material, which is available to authorized users.

## Introduction

The mycelial streptomycetes are a model organism for bacterial multicellularity (Claessen et al. 2014; Elliot et al. 2008). In the presence of sufficient nutrients, the soil-bound streptomycetes grow by tip extension and branching, producing an intricate network of vegetative hyphae to benefit optimally from the available nutrients. When the conditions become less favourable, *e.g.* nutrient deprivation (Rigali et al. 2008), activation of a morphological differentiation program (termed aerial development) resulting in the production of stress-resistant spores, is essential for survival and dissemination. The decision to enter aerial development is a critical and irreversible one, and is therefore tightly controlled (Chater 2001). The formation of aerial hyphae and spores is an energy-consuming process, whereby programmed cell death results in the dismantling of the substrate mycelium to provide nutrients to the new mycelium (Manteca et al. 2005, 2006). Streptomycetes are highly adapted to survive in diverse and complex ecosystems. This is highlighted by the presence in their genomes of more than 20 gene clusters specifying secondary metabolites, and genes encoding around 65 sigma factors and an unprecedented number of sugar transporters and polysugar hydrolases (Bentley et al. 2002; Cruz-Morales et al. 2013; Ohnishi et al. 2008). On solid-grown cultures, streptomycetes undergo a cycle of morphological development, whereby upon nutrient starvation aerial hyphae are formed on top of the vegetative mycelium. These aerial hyphae in turn undergo an extensive cell division event to produce chains of unigenomic spores (Chater 1972). Most of the developmental genes that control aerial development (the so-called *whi* genes) encode transcription factors (TFs) (Chater 1972; Ryding et al. 1999; Flärdh et al. 1999). More recently, it was shown that many genes involved in nutrient sensing and transport, such as *dasR, dasABC* and the *pts* genes, also control development and antibiotic production (Rigali et al. 2006; van Wezel et al. 2009).

The SsgA-like proteins are a family of proteins that control sporulation (Jakimowicz and van Wezel 2012; Traag and van Wezel 2008). Several *Streptomyces spp.* are capable of producing spores in liquid cultures (recently reviewed in (van Dissel et al. 2014)). SsgA was originally identified as a suppressor of a hyper-sporulating *S. griseus* mutant (designated SY1) and shown to be essential for submerged sporulation by this organism (Kawamoto and Ensign 1995; Kawamoto et al. 1997). Overexpression of SsgA in liquid-grown mycelium of *S. coelicolor* induces mycelial fragmentation and submerged sporulation (van Wezel et al. 2000). The ability of SsgA to enhance fragmentation and protein secretion was applied in industrial fermentations, revealing a significant improvement in yield and fermentation characteristics (van Wezel et al. 2006). The *ssgA, ssgB* and *ssgG* genes control the selection of septation sites in *S. coelicolor*, with *ssgA* and *ssgB* essential for sporulation (Keijser et al. 2003; Sevcikova and Kormanec 2003; van Wezel et al. 2000). SsgA dynamically controls the localization of its paralogue SsgB, which in turn recruits FtsZ to septum sites to initiate sporulation-specific cell division (Willemse et al. 2011). In mutants lacking *ssgG* septa are frequently skipped, resulting in many large spores containing multiple chromosomes that are well segregated (Noens et al. 2005).

Relatively little is known about how *ssg* gene expression is controlled. Transcription of *ssgA* has been studied in the model species *S. coelicolor* and *S. griseus*. A major difference between these two species, is that *S. griseus* sporulates in both surface- and liquid-grown cultures, while *S. coelicolor* only sporulates on solid media. The transcriptional control of early development differs significantly between these two species (Chater and Horinouchi 2003). The same is true for the transcriptional control of *ssgRA*; in *S. coelicolor*, transcription is activated by and dependent on SsgR (Traag et al. 2004), while in *S. griseus* transcription depends on the A-factor pathway-controlled AdpA, and only one of the two promoters is controlled by SsgR (Yamazaki et al. 2003). In submerged cultures of *S. coelicolor*, *ssgA* is poorly expressed (Romero et al. 2014). A recent study showed that the transcription of *ssgRA* and *ssgB* is controlled by the pleiotropic developmental regulatory protein BldD, providing an important connection between BldD and the control of sporulation-specific cell division (den Hengst et al. 2010). In this study, we further investigated the transcriptional control of *ssg* genes, including transcriptional dependency on the early *whi* genes *whiA*, *whiB*, *whiG*, *whiH*, *whiI* and *whiJ* as well as on *ssgB*. Transcriptional regulators that might directly control *ssg* transcription were identified using a DNA affinity capture assay. Of these, AtrA was shown to activate the transcription of *ssgR*, which in turn is required for the transcription of *ssgA*. This is the first developmental gene that has been identified as a target of AtrA.

## Materials and methods

### Bacterial strains and culturing conditions

*E. coli* K-12 strains JM109 (Sambrook et al. 1989) and ET12567 (MacNeil et al. 1992) were used for propagating plasmids, and were grown and transformed using standard procedures (Sambrook et al. 1989). *E. coli* BL21 (DE3) was used as host for protein production. Transformants were selected in Luria broth containing 1 % (w/v) glucose and the appropriate antibiotics.

The *Streptomyces* strains used in this work and their corresponding references are listed in Table S1. *S. coelicolor* A3(2) M145 is the parent of the mutants described in this work. The *atrA* (SCO4118), *rok7b7* (SCO6008), *slbR* (SCO0608) and *ssgB* (SCO1541) null mutants were described previously by the authors. Sporulation mutants *S. coelicolor* J2401 (Δ*whiA*), J2402 (Δ*whiB*), J2400 (Δ*whiG*), J2210 (Δ*whiH*), J2450 (Δ*whiI*) and C77 (*whiJ point* mutant C77) were obtained from the John Innes Centre strain collection. Preparation of media for growth, protoplast preparation and transformation of *Streptomyces* were done according to standard procedures (Kieser et al. 2000). As solid media we used SFM (soya flour mannitol) to make spore suspensions; R2YE (regeneration media with yeast extract) for regenerating protoplasts and, after addition of the appropriate antibiotic, for selecting recombinants; and minimal medium (MM) to prepare total RNA samples (Kieser et al. 2000). For standard cultivation of *Streptomyces* in liquid cultures we used YEME (yeast extract malt extract) containing 30 % (w/v) sucrose, TSBS (tryptone soy broth; Difco) containing 10 % (w/v) sucrose or NMMP (normal minimal medium buffered with phosphate) (Kieser et al. 2000). Microscopy was performed as described previously (Colson et al. 2008). Cultures were checked at regular intervals by phase-contrast microscopy using a Zeiss Standard 25 microscope and colony morphology was studied using a Zeiss Lumar V-12 stereo microscope.

### Plasmids and constructs

For routine subcloning, pIJ2925, a pUC19-derived plasmid, was used (Janssen and Bibb 1993). Plasmid DNA was isolated from ET12567 prior to transformation to *Streptomyces*. For selection of pIJ2925 in *E. coli*, ampicillin (100 µg/ml) was used; chloramphenicol (25 µg/ml) was added to select for growth of ET12567. The entire coding regions of the genes SCO0608 and SCO6008 were PCR-amplified from genomic DNA of *S. coelicolor* M145 using oligonucleotides described in Table S2; subsequently, they were cloned into expression vector pET24_ma_ (Novagen) using EcoRI and HindIII restriction sites, to allow the production of C-terminally hexahistidine-tagged versions of the corresponding proteins. The construct for the production of AtrA (encoded by SCO4118), which was N-terminally hexahistidine tagged, has been described previously (Uguru et al. 2005).

### RNA isolation and semi-quantitative and quantitative RT-PCR analyses

All oligonucleotides used for RT-PCR reactions are described in Table S2. For transcriptional analysis of *ssgA*-*ssgG* in surface-grown developmental (*whi*) mutants, mycelium grown on solid MM with mannitol (0.5 % w/v) on cellophane discs was harvested as indicated at three time points corresponding to vegetative growth, early aerial growth, and late aerial growth or, in the case of M145, spore formation. Phase-contrast light microscopy was used to assess the developmental stage of the surface-grown cultures prior to harvesting mycelium and isolating RNA. Semi-quantitative reverse-transcriptase PCR (RT-PCR) analysis was carried out using SuperScript III one-step RT-PCR System (Invitrogen) as described previously (Colson et al. 2007). For each RT-PCR reaction 200 ng of RNA was used together with 0.5 μM (final concentration) of each oligonucleotide. Samples were then analysed by electrophoresis using a 2 % agarose gel in TAE buffer. RT-PCR experiments without prior reverse transcription were performed on all RNA samples to assure exclusion of DNA contamination. As controls, mock reactions were carried out in the absence of the reverse transcriptase. Quantification of the RT-PCR results was done by scanning the gels using the GS-800 imaging densitometer followed by analysis using Quantity One software (Bio-Rad). 16S rRNA levels were analysed and quantified as a control, and values obtained for the *ssg* genes were corrected for slight differences in the 16S rRNA levels in the corresponding RNA extracts.

Quantitative PCR analysis was carried out following reverse transcription that used SuperScript II, as instructed by the vendor (Invitrogen), in combination with 500 ng of DNase I-treated total RNA as template and 50 ng of random hexamers (Applied Biosystems) as primer in 20 µl reactions. Mock reactions without the reverse transcriptase were also conducted. At the end of each of the reactions, 80 µl of yeast tRNA (10 ng/ml; Ambion) was added, and 2 µl aliquots analysed by PCR using a SensiMix™ SYBR No-ROX kit, as instructed by the vendor (Bioline), in combination with 0.5 µM primers and 3.5 mM MgCl_2_. Reactions were assembled in 0.1 ml strip tubes and analysed using a real-time cycler (Corbett Rotor-Gene 6000). The PCR conditions were 10 min at 95 °C, followed by 40 cycles of 10 s at 95 °C, 15 s at 60 °C and 15 s at 72 °C. The products were analysed by determining melting curves and confirming the sizes of the amplicons by gel electrophoresis. A threshold common to the exponential phases of all the reactions in a single run was selected manually and the corresponding number of cycles for each reaction recorded. These CT values were then expressed as the difference relative to *rpsL* (SCO4735), which was used as the internal control. In turn, the ΔCT values were used to calculate the difference in abundance using the Equation 2^ΔCT^.

### DACA assay

The DACA procedure was described previously (Park et al. 2009). In brief, streptavidin Dynabeads (Dynal Biotech, Oslo, Norway) were washed and incubated with annealed oligonucleotides (100 pmol DNA/mg of beads) for 30 min at room temperature, and biotin (100 μg/ml) was subsequently added and further incubated for 15 min. 1 ml of a pre-mixed cell extract solution (500 μg/ml) containing sheared salmon sperm DNA (0.1 mg/ml) was incubated for 15 min on ice and then added to 100 μl of bead solution (0.5 mg beads and 0.1 mg cellular protein). Following 40 min incubation at room temperature, the beads were washed with standard buffer and then resuspended in 100 mM NH_4_HCO_3_ (pH 8). The captured protein mixtures were then digested with trypsin, and tryptic peptides were analysed by liquid chromatography-tandem mass spectrometric analysis (LC–MS/MS) using an LTQ-orbitrap mass spectrometer (Thermo Finnigan/Thermo Fisher, Waltham, MA, USA). Peptide ions were detected in full scan mode from 400 to 1700 m/z followed by three data-dependent MS/MS scans (isolation width 1.5 m/z, 35 % normalized collision energy, dynamic exclusion for 5 min) in a completely automated fashion.

Proteins were identified by searching the MS/MS spectra against a *S. coelicolor* protein database using SEQUEST (Eng et al. 1994) with Sequest Sorcerer software (Thermo Scientific). An XCorrelation score was calculated based on the cross-correlation peptide score following a SEQUEST database search (Eng et al. 1994). The following criteria were used to sort out proteins from MS spectra. The cut-off for the cross-correlation score (Xcorr) was >1.7 for +1 charged tryptic peptides, >2.5 for +2 charged peptides, or >3.0 for +3 charged peptides. The delta correlation value (ΔCn) was at least 0.15, regardless of the charge state (Ducret et al. 1998). Finally, only those hits were considered for which at least two peptides were identified with a significant Xcorrelation score.

### Protein production and purification

To produce recombinant hexahistidine-tagged proteins, cultures of *E. coli* BL21 (DE3) harbouring pET24-SCO0608 or pET24-SCO6008 were grown in 1 l of LB broth at 37 °C until an OD_600_ of 0.6–0.8, and induced by the addition of IPTG to 0.5 mM. Extracts were prepared and proteins purified using a column packed with Nickel-NTA agarose resin (BioRad) essentially as described (Mahr et al. 2000). Columns were washed with 10 column volumes of binding buffer (50 mM sodium phosphate buffer; 300 mM NaCl; 0.01 % Tween20) and proteins eluted with three column volumes of elution buffer (binding buffer containing 100 mM imidazole). The eluted proteins were desalted and concentrated using 15-ml centrifugal filter unit (Millipore, molecular weight cut-off of 10 kDa). The concentrated protein extracts of ~300 μl volume were immediately used or flash-frozen with liquid N_2_ for storage at −80 °C.

### Electrophoretic mobility shift assay (EMSA)

To obtain probes for EMSAs, DNA fragments containing the upstream regions of the target genes were amplified by PCR and purified, following electrophoresis, from agarose gels. For EMSAs with SlbR, PCR products were end-labelled with [γ-32P] dATP using T4 polynucleotide kinase and purified using Probe-Quant™ G-50 microcolumns (GE Healthcare, USA). The labeled probe and purified recombinant protein of different concentrations were added to a solution containing 0.5 × TBE (pH 8.0), 0.0375 % (w/v) glycerol, 5 mM MgCl_2_, 1 mM DTT, and 7.5 μg sheared salmon sperm DNA (20 μl final volume). After 30 min incubation at room temperature, 1 μl of 10 % glycerol was added to each mixture and samples were immediately loaded onto a 4 % non-denaturing polyacrylamide gel, which was subjected to electrophoresis in 0.5 × TBE. Gels were dried under vacuum and exposed to phosphor-screen and images acquired using a Typhoon 8600 scanner (GE Healthcare). EMSAs with AtrA were done as described previously (Uguru et al. 2005), except the probes were fluorescently labelled via the incorporation of a FAM group at the 5′ end of one of the PCR primers during its synthesis (Eurofins). The 224-bp *ssgR* probe (−321 to −97 relative to the start of the gene) was amplified using primers ssgRp1 and ssgRp2, the 100-bp *act*II-ORF4 (site 2) probe (+115 to +15) was amplified using actII-4(s2)p1 and actII-4(s2)p2, and the 179-bp SCO4119 probe (−305 to −126) was amplified using SCO4119p1 and SCO4119p2.

## Results

### Transcription of *ssg* genes in sporulation mutants of *S. coelicolor*

**Fig. 1:**
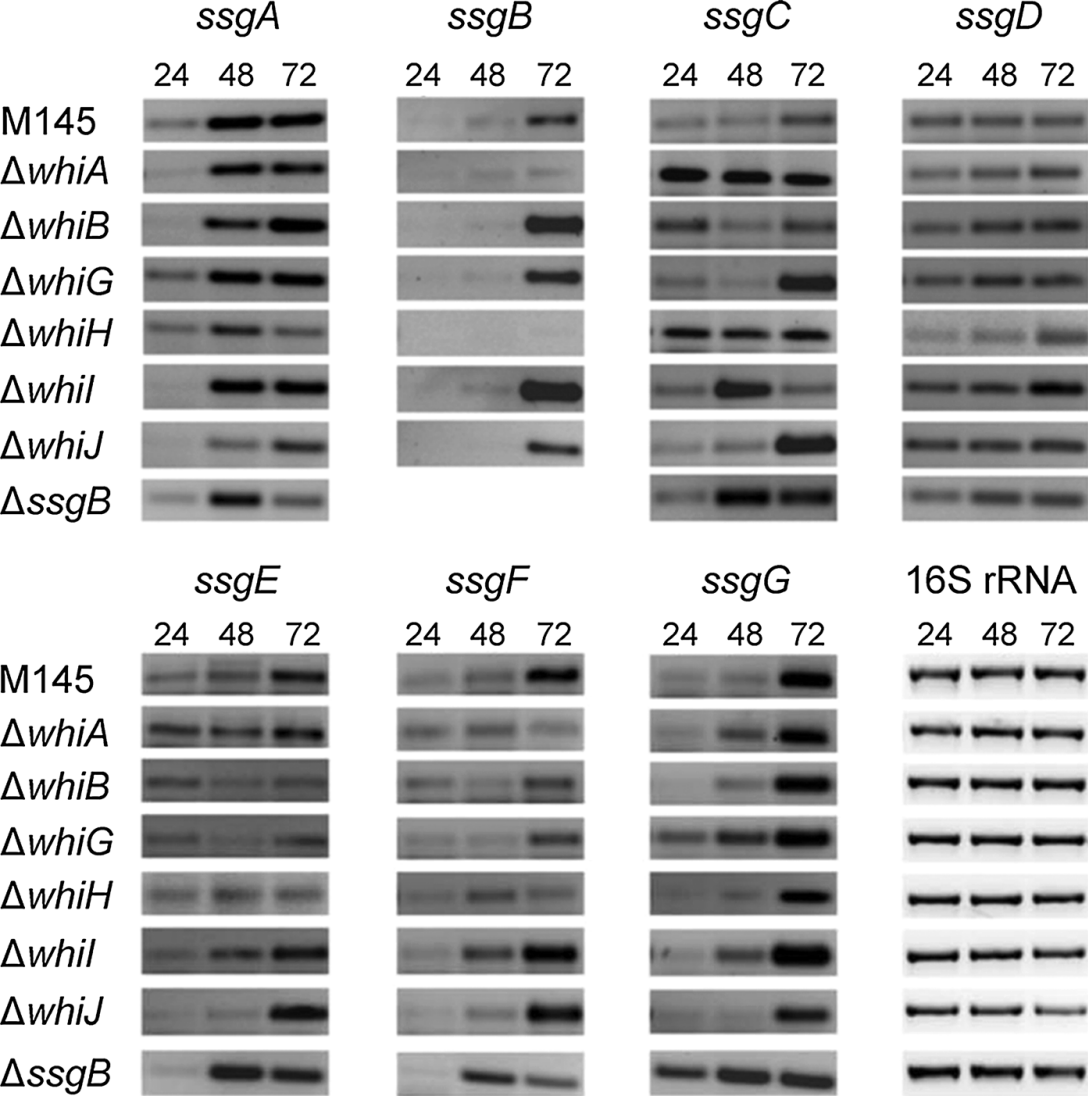
Transcription of *ssg* genes in *whi* mutants of *S. coelicolor*. Semi-quantitative RT-PCR data of *ssgA*-*G* and 16S rRNA (*vertical axis*) performed on RNA purified from the parental strain M145 and its *whiA*, *whiB*, *whiG*, *whiH*, *whiI*, *whiJ* and *ssgB* mutants (*horizontal axis*). Time points: 24 h (vegetative growth), 48 h (aerial growth) and 72 h (late aerial growth and where relevant sporulation) of growth on MM agar plates, respectively

We have previously shown that *ssgRA* are transcribed independently of the sporulation genes *whiA*, *whiB*, *whiG*, *whiH*, *whiI* and *whiJ* in *S. coelicolor* (Traag et al. 2004). Here we expanded this survey so as to include the transcriptional analysis of *ssgB*-*G* in the genetic background of these ‘classical’ *whi* mutants as well as in *ssgB* mutants (Keijser et al. 2003). For this, semi-quantitative RT-PCR was done on RNA isolated from mycelia grown for 24, 48 or 72 h on MM agar plates with mannitol as the sole carbon source. After 24 h, *S. coelicolor* M145 produced a vegetative mycelium, aerial hyphae were formed after 48 h and abundant sporulation was seen after 72 h. 16S rRNA was used as the control. The results are shown in Fig. 1. RT-PCR data were quantified and corrected for loading differences using 16S rRNA as an internal reference (see Materials and Methods section). In the parental strain *S. coelicolor* M145 transcription of *ssgA*, *ssgB*, *ssgC*, *ssgE*, *ssgF* and *ssgG* was life cycle-dependent, with increased transcript levels at stages in development, while *ssgD* transcript levels were equally high at all stages (Fig. 1). As reported earlier, *ssgA* transcription was not significantly (less than two-fold) altered in any of the *whi* mutant backgrounds (although *ssgA* transcript levels appeared somewhat reduced in the *whiH* mutant), and the same was true for *ssgD*. Transcription of the cell division gene *ssgB* was primarily seen during late development (72 h), which corresponds to sporulation in wild-type cells. Transcription of *ssgB* was more than three-fold reduced in the *whiA* mutant, and nearly absent in the *whiH* mutant. Transcription of *ssgG*, a functional homologue of *ssgB*, was also strongly development-dependent, and reduced in the *whiH* mutant, as well as in the *whiJ* mutant. Surprisingly, transcript levels of *ssgG* appeared constitutive in an *ssgB* mutant. Differential expression of other *ssg* genes was seen in several sporulation mutants: transcription of *ssgC* was enhanced in one or more time points of most of the *whi* mutants and in the *ssgB* mutant, while the spore-maturation genes *ssgE* and *ssgF* were upregulated moderately in *whiI, whiJ* and *ssgB* mutants.

### Screening for novel transcription factors that control *ssgR, ssgA*, *ssgB* and *ssgG*

To identify TFs that may directly control the transcription of the sporulation-specific *ssg* genes, we carried out DNA affinity-capture assays (DACA; (Park et al. 2009)) using biotinylated DNA baits corresponding to the promoter regions of *ssgA*, *ssgB*, *ssgG* and *ssgR*. For details on method and significance of the hits, see Materials and Methods section. *S. coelicolor* M145 was grown in YEME media, samples were collected after 48, 72 and 100 h of growth, and total cell extracts prepared. Probes were washed extensively and bound proteins identified by high sensitivity mass spectrometry. The power of the DACA method is that DNA-bound proteins are identified at low concentrations, but the sensitivity of mass spectrometry also means that subsequent validation by EMSAs with purified candidate proteins is required. DACA assays identified ten different proteins in the various fractions containing proteins that bound to at least one of the *ssg* promoter regions, all of which are known or predicted DNA binding proteins (Table 1 and Table S3); this indicates significant specificity for the method used, considering that the *S. coelicolor* genome encodes nearly 1000 proteins with predicted regulatory function (Bentley et al. 2002). These 10 proteins were SCO0608 (SlbR), SCO1839, SCO2792 (AdpA), SCO2950 (HupA), SCO3198 (FruR), SCO3375, SCO3606, SCO3859, SCO4118 (AtrA) and SCO5803. Of these, SCO1839, SCO3375 and AtrA were specifically identified for only one of the *ssg* promoter fragments. Two proteins were found for all probes, namely HupA, one of the three nucleoid-associated HU-homologous proteins produced in vegetative hyphae (Salerno et al. 2009) and a general DNA-binding protein, and FruR, which most likely controls the fructose metabolic operon *fruKA* encoding the fructose kinase (SCO3197) and fructose permease (SCO3196) (Nothaft et al. 2003).

**Table 1:** Proteins binding to the *ssg* probes as identified by DACA

Grey shading means significant binding, with a minimum of two identified peptides per hit and significant Xcorrelation scores in SEQUEST (Eng et al. 1994). For details see Table S3 and “Materials and methods” section

### AtrA directly controls *ssgR* transcription

**Fig. 2:**
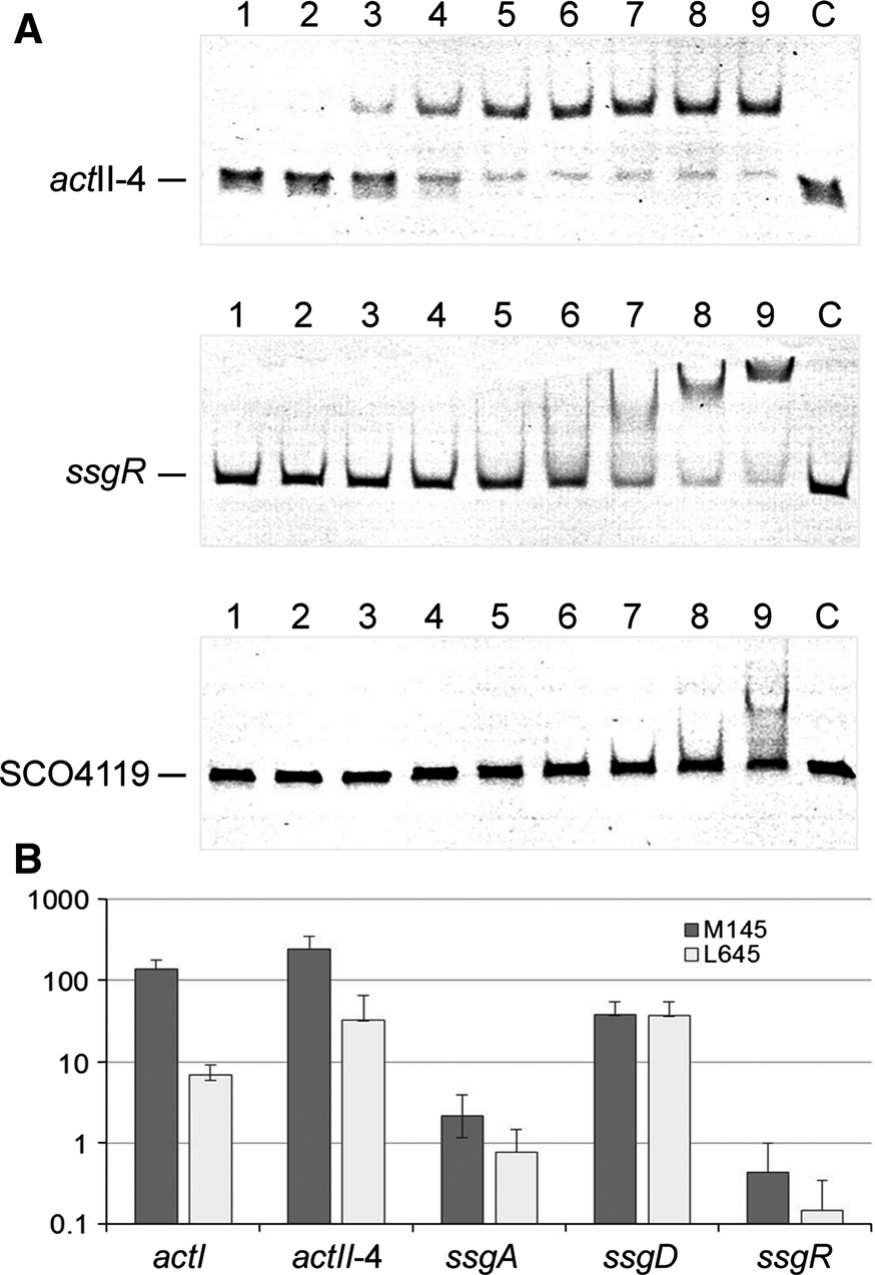
Analysis of AtrA regulation of *ssgR.* **A** Binding of recombinant AtrA to the promoter region of *ssgR* in vitro. Binding to Site 2 within the promoter region of *act*II-ORF4 and the AtrA-controlled promoter of SCO4119 were included as controls. Labelling on the left identifies the probes. *Lanes* 1–9 contain 19, 38, 75, 150, 300, 600, 1250, 2500 and 5000 nM of AtrA (dimer), respectively. *Lane* C contains no AtrA. The concentration of DNA probe in each reaction was 5 nM. **B** Dependency of transcript levels on *atrA* in vivo. Histogram showing the average levels of transcripts in M145 and L645 (the congenic Δ*atrA* partner of M145) expressed as a percentage of the average abundance of the *rpsL* transcript (SCO4735). The values are the average of three independent measurements. *Bars* indicated the standard error. The *Y axis* has a log scale

The DACA technology identified AtrA as possible regulator for *ssgR*. AtrA is a TetR-family transcriptional regulator that in *S. coelicolor* directly activates *act*II-ORF4 (Uguru et al. 2005) and *nagE2* (Nothaft et al. 2010), which encode the pathway-specific activator for actinorhodin biosynthetic gene cluster and the membrane component of the N-acetylglucosamine transporter, respectively. Analysis using the PREDetector algorithm (Hiard et al. 2007) identified a likely AtrA responsive element (*are*), namely the inverted repeat sequence GGAACCACCGGTTCC (complementary sequences underlined), corresponding to nt positions −166/−152 relative to the *ssgR* translational start (see Discussion). This upstream element conforms very well to the consensus sequence published previously (Nothaft et al. 2010). To test whether this putative *cis*-acting element is indeed recognized specifically by AtrA, we performed electrophoretic mobility shift assays (EMSAs) using purified recombinant AtrA (Uguru et al. 2005). Comparison with previous results was facilitated by including in the analysis fragments containing the AtrA-binding site within the coding region of *act*II-ORF4 (Uguru et al. 2005) and the recently reported AtrA-controlled promoter of SCO4119 (encoding a putative NADH dehydrogenase) (Ahn et al. 2012), which is divergent from the *atrA* promoter (SCO4118). As reported previously, AtrA bound within the coding region of *act*II-ORF4 with an apparent equilibrium dissociation constant of *K*_*d*_*’* of 150 nM (Uguru et al. 2005). AtrA also bound to the *ssgR* promoter (Fig. 2a), albeit that the affinity of this interaction was 8-fold weaker (*K*_*d*_*’* of 1.2 µM). It was however stronger than the binding of AtrA to the promoter of SCO4119.

The effect of AtrA on the expression of *ssgR* was investigated using quantitative RT-PCR (qPCR) analysis of transcript abundance in M145 and L645, the congenic *atrA* knockout (Uguru et al. 2005). To permit several transcripts with a range of abundances to be included in the study, we adopted a quantitative approach (for details, see Materials and Methods). After preliminary investigations, *rpsL*, which encodes ribosomal protein S11, was adopted as the internal control because it is constitutively expressed and the abundance of its mRNA is in the middle of the range for transcripts of interest (Romero et al. 2014). Furthermore, the analysis focused on mycelia grown on MM agar plates with mannitol as the sole carbon source to the point at which aerial hyphae began to emerge. This offered excellent discrimination of the effects of *atrA* on *act*II-ORF4 and members of its regulon (Fig. 2b). The average fold decreases in the abundance of the transcripts of *act*II-ORF4 and *act*I in L645 were 20.1 (±1.9) and 7.5 (±2.9), respectively. These data are in line with our previous data showing that transcription of the *act* biosynthetic gene cluster depends on AtrA (Uguru et al. 2005). The level of the *ssgD* transcript was unchanged (1.0 ± 0.1). In contrast, the level of the transcript of *ssgR* and the transcript of *ssgA*, the target of SsgR activation, decreased on average 2.9 (±0.5) and 2.8 (±0.7) fold, respectively. These qRT-PCR results provide in vivo evidence consistent with the direct transcriptional activation of *ssgR* by AtrA. Moreover, the overall analysis described in this section provides strong validation for the applicability of DACA. It also should be noted that AtrA binding to the *act*II-ORF4 promoter was detected by the DACA assay (data not shown). The considerably higher abundance in M145 of the *ssgD* transcript relative to those of *ssgA* and *ssgR* (Fig. 2b) was validated by independent promoter probing results (unpublished data). The level of the *ssgD* transcript, as well as those of *act*II-ORF4 and *act*I, was similar to that of *rpsL*, which encodes one of the most abundant proteins in bacteria.

### The γ-butyrolactone receptor protein SlbR binds upstream of *ssgA, ssgB* and *ssgR*

**Fig. 3:**
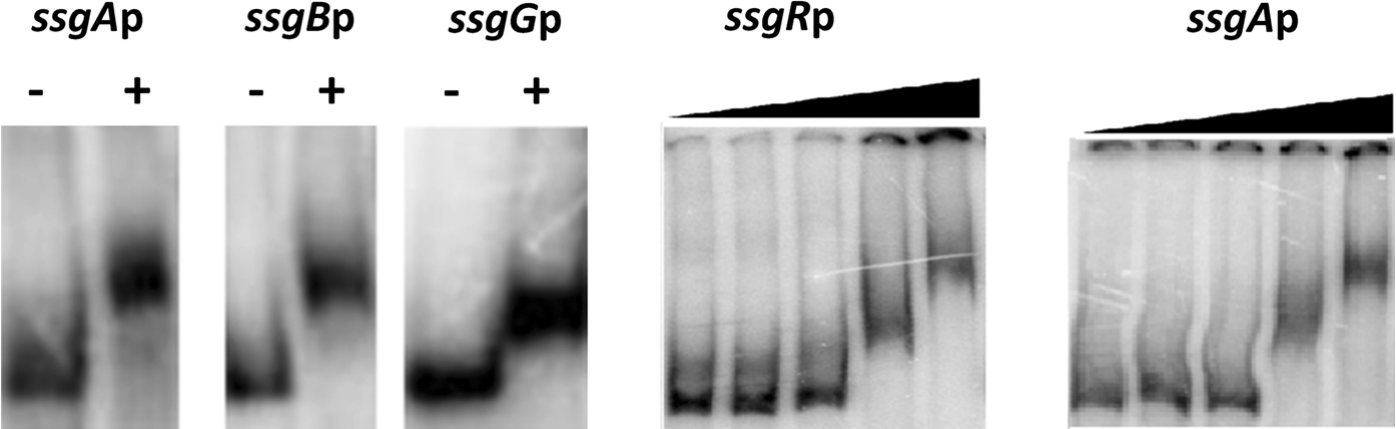
EMSAs showing binding of SlbR to the *ssg* target sequences in vitro. *Left*, binding of purified SlbR-His_6_ (3.5 μg) to ^32^P-radio-labeled probes of *ssgA, ssgB*, and *ssgG* upstream sequences. *Right*, Binding of SlbR-His_6_ to the upstream sequence of *ssgR* and *ssgA*. DNA was incubated with (from *left* to *right*) no SlbR protein (control), 0.1, 0.4, 3.4, and 6.8 μg protein

The DACA assays identified two γ-butyrolactone-responsive proteins, AdpA and SlbR (for ScbR-like γ-butyrolactone binding regulator; SCO0608), as potential regulators of multiple *ssg* genes. This connects to earlier data showing that AdpA is required for transcription of *ssgA* in *S. griseus* (Yamazaki et al. 2003). However, deletion of *adpA* has little effect on *ssgA* transcription in *S*. *coelicolor* (Traag et al. 2004). SlbR is a receptor for the γ-butyrolactone Scb1 of *S. coelicolor* and binds to the *scbRA* and *adpA* promoter regions, but does not share significant sequence homology to the canonical γ-butyrolactone receptor protein ScbR in *S. coelicolor* (Yang et al. 2012). To investigate if indeed SlbR binds to the promoter regions of the *ssg* genes, EMSAs were performed, whereby all four *ssg* promoter regions were tested as probes (Fig. 3). This revealed direct binding of recombinant SlbR to the upstream regions of *ssgA, ssgB*, *ssgG* and *ssgR*, albeit at higher protein concentrations (μM range), suggesting that the affinity for the promoters in vitro is relatively weak. The binding site for SlbR is yet unknown (Yang et al. 2012), and similarity searches using the promoter regions for the *ssg* genes and the known SblR target *scbR* failed to identify a common regulatory element (not shown). Preliminary transcript analysis on surface-grown cultures by qRT-PCR did not reveal major differences in expression of *ssgA, ssgB*, *ssgG* or *ssgR* in the *slbR* null mutant (unpublished data). It should be noted that the DACA assays were done with extracts obtained from liquid-grown cultures, which may explain this apparent discrepancy.

## Discussion

Streptomycetes are multicellular bacteria that undergo an unusually complex life cycle among microorganisms, and while gradually more regulatory networks are being uncovered, the number of key developmental genes whose function has been elucidated in detail is still rather limited (Chater et al. 2010; Flärdh and Buttner 2009). Well-studied developmental genes are the *whi* genes, which encode different types of regulatory proteins (Chater 2001), and the *ssg* genes, for members of the family of SsgA-like proteins (SALPs) that do not control transcription, but instead likely act as chaperone-like proteins that mediate processes related to peptidoglycan synthesis and remodeling (Noens et al. 2005). To establish possible connections between the *whi* and *ssg* networks, we have looked into *ssg* gene expression in mutants lacking one of the early *whi* sporulation genes *whiA*, *whiB*, *whiG*, *whiH*, *whiI*, and *whiJ*, and identified regulatory proteins that control transcription of the cell division regulatory genes *ssgA, ssgB, ssgG* and *ssgR*.

Transcription of the key sporulation regulatory gene *ssgB* was strongly down-regulated in *whiA* mutants and nearly abolished in *whiH* mutants. These data conform well to recent microarray analyses, which showed that transcription of both *ssgB* and *ssgG* depends on *whiA* and *whiH* (Salerno et al. 2013). The transcription of *ssgG*, which likely is a functional homologue of *ssgB* (Girard et al. 2013), was also reduced significantly in *whiH* mutants, but under the chosen growth conditions we did not see a decrease in *ssgG* transcription in *whiA* mutants. Interesting parallels exist between the developmental phenotypes of *S. coelicolor* colonies lacking either *ssgB* or *whiA*. Mutants of *whiA* and *ssgB* have white (non-sporulating) phenotypes, producing aseptate aerial hyphae and no spores (Ryding et al. 1999; Flärdh et al. 1999; Keijser et al. 2003). Furthermore, *whiA* and *ssgB* mutants both appear to lack the signal for aerial growth arrest that precedes the onset of sporulation (Chater 2001), with *whiA* mutants producing very long aerial hyphae, while colonies of the *ssgB* mutant have a large colony phenotype (Flärdh et al. 1999; Keijser et al. 2003). The precise connection between WhiA and SsgB requires further investigation.

Similar to *ssgRA* (Traag et al. 2004), transcription of *ssgD* was not significantly affected in the six early *whi* mutants or in an *ssgB* mutant. This is perhaps not surprising, as SsgD is the only SALP that plays a role during vegetative growth: the *ssgD* gene is strongly transcribed during vegetative growth, and *ssgD* null mutants have pleiotropic defects in the lateral wall of the vegetative hyphae (Noens et al. 2005). A logical explanation for the *whi*-independent expression of *ssgA* is that it also plays a role in germination and branching, which occur during vegetative growth (Noens et al. 2007), and similar arguments can be used for *ssgD*. Finally, transcription of *ssgC*, whose function is less well understood, and of *ssgE* and *ssgF*, which are involved in spore maturation (Noens et al. 2005), was deregulated to some extent in most of the *whi* mutants as well as in the *ssgB* mutant.

Besides the control of *ssgB* and *ssgG* by WhiA and WhiH, the roles of the Whi and SALP proteins in development appear primarily parallel. Indeed, the Whi regulatory proteins ensure the correct timing of the transcription of *ftsZ* during sporulation, whereby accumulation of FtsZ is a major prerequisite for the onset of sporulation-specific cell division (Flärdh et al. 2000; Willemse et al. 2012). Conversely, the SALPs primarily play a role in the control of peptidoglycan remodeling, such as during cell division and spore-wall synthesis. More detailed information on the Whi regulatory networks is required to provide further insights into their precise regulatory networks.

### Novel regulators of *ssg* gene expression

DACA assays combining affinity chromatography with MALDI-ToF mass spectrometry and using the promoter regions for *ssgA, ssgB, ssgG* or *ssgR* as baits, identified in total ten candidate regulatory proteins that bound to one or more of the *ssg* promoters (Table 1). The fact that all proteins were indeed annotated DNA binding proteins, and that over 1000 regulatory proteins are encoded by the *S. coelicolor* genome, provides significant validation for the assay. Of the ten proteins, HupA is a ‘general’ DNA-binding protein belonging to the family of nucleoid-associated HU- proteins (Salerno et al. 2009).

Two regulators involved in A-factor mediated quorum sensing, AdpA (SCO2792) and SlbR (SCO0608), and an activator for antibiotic production, AtrA (SCO4118), were also identified. Of these, AdpA is known to control *ssgA* transcription in *S. griseus* (Yamazaki et al. 2003), suggesting that AdpA is indeed a *bona fide* hit in the DACA assay. However, unlike for the model system *S. griseus*, the AdpA regulon of *S. coelicolor* is not well known, and direct development-related targets were identified only recently (Wolanski et al. 2011), namely *sti*, encoding a protease inhibitor (Kim et al. 2008), and *ramR*, for an atypical response regulator that activates the expression of the small modified peptide SapB required for the onset of aerial growth (Willey and Gaskell 2011; Nguyen et al. 2002; Keijser et al. 2002). SlbR was recently identified as a novel γ-butyrolactone receptor-like protein in *S. coelicolor*, with apparently similar behaviour as ArpA in *S. griseus* (Yang et al. 2012). Although both regulators affect antibiotic production and spore formation, none of the studies previously related the sporulation-specific SALPs to these autoregulator-controlled regulators. While EMSAs showed that SlbR binds to the promoter region of all *ssg* genes analysed (*ssgA, ssgB, ssgG and ssgR*), preliminary transcript analysis did not reveal significant changes in *ssg* transcript levels in the *slbR* null mutants grown on SFM agar plates. However, the DACA assays were done using extracts obtained from liquid-grown cultures, and it is difficult to compare the two growth conditions. While in the function of SsgB has only been studied in surface-grown cultures (*i.e.* in the aerial hyphae), its expression is also strongly enhanced during late exponential growth (Huang et al. 2001). Besides recruiting FtsZ during sporulation-specific cell division, SsgB also plays a role in growth cessation, with *ssgB* null mutants displaying a large colony phenotype (Keijser et al. 2003). Furthermore, many streptomycetes sporulate in submerged cultures (Girard et al. 2013), a process that is mechanistically very similar to aerial sporulation and requires SsgA (Kawamoto et al. 1997) and most likely also SsgB. The effect of SlbR on the control of growth and sporulation of streptomycetes remains to be analyzed in more detail.

A conspicuous DACA hit was the TetR-family transcriptional regulator AtrA. AtrA directly activates *actII*-ORF4, encoding the pathway-specific transcriptional activator for the actinorhodin biosynthetic gene cluster in *S. coelicolor* (Uguru et al. 2005), and modulates the expression of *strR*, the pathway-specific transcriptional activator of streptomycin biosynthesis in *S. griseus* (Hirano et al. 2008; Hong et al. 2007). Mutants deleted for *atrA* fail to develop, and the reason for this developmental blockage has so far remained unexplained. We here show that AtrA activates the transcription of the sporulation regulatory gene *ssgR*. AtrA was shown to bind specifically to sequences in the upstream region of *ssgR*, and analysis using the PREDetector algorithm (Hiard et al. 2007) identified a likely AtrA-responsive element (*are*), namely the inverted repeat sequence GGAACCACCGGTTCC (−289 to −275 relative to the predicted start of *ssgR*; complementary sequences underlined). It should be noted that the predicted translational start of *ssgR* is not correct and is most likely the TTG triplet 123 nt upstream (start at chromosomal position 4318125), since proteomics experiments have unequivocally shown that the SsgR protein is at least 19 aa longer than predicted. The SsgR protein is thus extended by the aa sequence: LATADTAFAQLHALPVQPCPTSRTPPASPSADPPSATLIGSV, with the underlined residues identified by mass spectrometry (our unpublished data). In addition to identifying AtrA binding to the promoter region of *ssgR*, disruption of *atrA* in vivo strongly reduced transcription of *ssgR* and *ssgA*.

Taken together, our data strongly suggest that AtrA activates the transcription of *ssgR*, the gene product of which is in turn required for the transcriptional activation of *ssgA*. It has always been a bit of a mystery as to why *ssgRA* should not be influenced by any of the Whi sporulation genes and what the alternative mechanism is. The likely explanation for the former is that SsgA is not only needed for sporulation-specific cell division, but also is active at a time when the Whi proteins are not yet active, namely during germination and tip growth and branching of the vegetative hyphae (Noens et al. 2007). We now show that AtrA is a major regulator of *ssgRA* gene expression. Also, this is the first developmental gene that was shown to be controlled by AtrA and offers an explanation as to why sporulation is impaired in the *atrA* null mutant. However, since *ssgRA* are only required for sporulation, this does not explain why AtrA is required for aerial hyphae formation.

### Concluding remarks

This study expands our insights into how in particular the cell division-related *ssg* genes are controlled during development. Multiple TFs control the sporulation genes *ssgA* and *ssgB*, which ultimately control the onset of sporulation-specific cell division. DACA combined with transcript analysis showed that *ssgR* transcription is directly activated by the developmental regulator AtrA, which also activates actinorhodin biosynthesis. This provides a novel and direct link between development and antibiotic production, and also highlights the first developmental target for AtrA itself. In *S. griseus*, AdpA controls *ssgA* transcription, and the DACA suggested that AdpA may indeed control *ssgA*. Binding of SlbR was seen for the *ssgA, ssgB* and *ssgG* upstream sequences and was validated by EMSAs, but under the conditions chosen, we did not see a major effect on *ssg* gene expression in the *slbR* null mutant. Finally, several other TFs were identified as possible regulators of the *ssg* genes, and the precise role of these TFs in the control of *ssg* gene expression and in the control of cell division and development of streptomycetes should be analysed further.

## Electronic supplementary material

Supplementary material 1 (PDF 212 kb)
