# Supplementary material for: Transcriptional analysis of the cell division-related ssg genes in Streptomyces coelicolor reveals direct control of ssgR by AtrA
Source: Antonie Van Leeuwenhoek. 2015 May 23;108(1):201–13. doi: 10.1007/s10482-015-0479-2 (PMC4457907; doi:10.1007/s10482-015-0479-2)
Supplement: Supplementary file 1 — Supplementary material 1 (PDF 212 kb) [file 10482_2015_479_MOESM1_ESM.pdf]

Transcriptional analysis of the cell division-related *ssg* genes in *Streptomyces coelicolor* reveals direct control of *ssgR* by AtrA

Songhee H. Kim[1, *], Bjørn A. Traag[2, *], Ayad H. Hasan[3, *], Kenneth J. McDowall[3], Byung-Gee Kim[1] and Gilles P. van Wezel[4, #]

[1] School of Chemical and Biological Engineering and Institute of Molecular Biology and Genetics, Seoul National University, Kwanak-gu, Seoul 151-744, Korea;

[2] Bayer CropScience LP, Biologics, 890 Embarcadero Drive, West Sacramento, California, 95605, USA;

[3] Astbury Centre for Structural Molecular Biology, Faculty of Biological Sciences, University of Leeds, Leeds, LS2 9JT, United Kingdom;

[4] Molecular Biotechnology, Institute of Biology, Leiden University, PO Box 9505, 2300RA, Leiden, The Netherlands;

* these authors contributed equally to the work.

[#] To whom correspondence should be addressed. Tel: +31 71527430; email: g.wezel@biology.leidenuniv.nl

Table S1. *Streptomyces* strains used in this study.

**Strains**	**Description**	**Reference**
S. coelicolor M145	Wild type *S. coelicolor* A3(2)	(Kieser *et al*., 2000)
S. coelicolor J2401	M145 Δ*whiA*	(Flärdh *et al*., 1999)
S. coelicolor J2402	M145 Δ*whiB*	(Flärdh *et al*., 1999)
S. coelicolor J2400	M145 Δ*whiG*	(Flärdh *et al*., 1999)
S. coelicolor J2210	M145 Δ*whiH*	(Ryding *et al*., 1999)
S. coelicolor J2450	M145 Δ*whiI*	(Ainsa *et al*., 1999)
S. coelicolor C77	M145 *whiJ* locus 77	(Ryding *et al*., 1999)
S. coelicolor L645	M145 Δ*atrA*	(Uguru *et al*., 2005)
S. coelicolor GSB1	M145 Δ*ssgB*	(Keijser *et al*., 2003)
S. coelicolor	M145 Δ*slbR*	(Yang *et al*., 2012a)

Table S2. Oligonucleotides used in this study.

Primer name	DNA sequence (5' to 3')*	5' end	Relative to
Primers for semi-quantitative RT-PCR			
PssgA-for	GTC*GAATTC*CACCATGGCGCGCTGGCGCGAC	-619	*ssgA*
PssgA-rev	CTG*GGATCC*CCCGGGTCTCGTAGCGCAGCTC	+70	*ssgA*
PssgR-for	GTC*GAATTC*GGACTGCCGTGGTGGGTGAAGTG	-512	*ssgR*
PssgR-rev	GTC*GGATCC*CGCCCGCTGCACGGAGCCGATC	+12	*ssgR*
PssgB-rev	GTCC*GAGCTC*GCTCTCCCGAGTGATCACTGGTC	-592	*ssgB*
PssgB-for	GTC*GGATCC*GCAGCTGACCGTGGTGTTGAT	+21	*ssgB*
PssgC-rev	GTC*GGAATTC*GTCGACGCCGGGTTCACCGAGGT	-216	*ssgC*
PssgC-for	GTC*GGATCC*TGCACGACCAGGGTCTTGTGCAC	+35	*ssgC*
PssgD-for	GTC*GAATTC*GTCCCGTGCGTCGCGTGCTTCCC	-291	*ssgD*
PssgD-rev	GTC*GGATCC*ACTGCTCGATGACGGTGGAC	+22	*ssgD*
PssgE-for	GTC*GAATTC*GAGGTCGGGGCGTTGATGAATC	-223	*ssgE*
PssgE-rev	GTC*GGATCC*AGGATGTGGGCTCGTGCGTAC	+38	*ssgE*
PssgF-for	GTC*GAATTC*CGCGTGGGCCTGACCGGACATGAC	-401	*ssgF*
PssgF-rev	GTC*GGATCC*CTCGAGAGCGCCCGTCATCTG	+99	*ssgF*
PssgG-for	GTC*GAATTC*CTCGACCGGGTCCTCGTCGAAG	-467	*ssgG*
PssgG-rev	GTC*GGATCC*AGGACGAGCCTGAGCTCCAG	+62	*ssgG*
Primers for qRT-PCR (atrA mutant)			
SCO3926p1	GACCGCACCGACAAGCTG	+297	*ssgA*
SCO3926p2	GCTCTGTTCCTCCGCCAG	+401	*ssgA*
SCO6722p1	GGGAGAAGGTCCAGCAGA	+171	*ssgD*
SCO6722p2	GCCACCCTGCACTACGAC	+72	*ssgD*
SCO3925p1	GCTGTTCTTCCTCGGTGA	+155	*ssgR*
SCO3925p2	CCGGTACGTGGCGTAGTA	+290	*ssgR*
SCO5085p1	TACTTCGCCCTGATCGAC	+279	*act*II-ORF4
SCO5085p2	CTCGCCAGAGATCCAGTC	+417	*act*II-ORF4
SCO4735p1	GGCAAGTGGAAGGTCAAC	+192	*rpsL*
SCO4735p2	GTCGTAGCGGTTGTCCAG	+302	*rpsL*
SCO5087p1	CAACGCGTACCACATGAC	+938	*act*I
SCO5087p2	GTGCGCGTTGATGTAGTC	+1055	*act*I
Primers for qRT-PCR (slbR mutant)			
5S rRNA_RTL	GTCATAGCGTAGGGGAAACG	+11	5S rRNA
5S rRNA_RTR	GGCGTCCTACTCTCCCACAG	+104	5S rRNA
ssgA_RTL	TGTCCGAGGAGCTCTCTTTC	+17	*ssgA*
ssgA_RTR	GACCTGAAGTCGGATCAGCA	+243	*ssgA*
ssgB_RTL	CTCGTTGTGTCGAGCGAGT	+36 [a]	*ssgB*
ssgB_RTR	AGCTGAGAGCGATGCACAC	+267 [a]	*ssgB*
ssgG_RTL	AGCTCAGGCTCGTCCTGTC	+46	*ssgG*
ssgG_RTR	GTCTTCGACGGCCACACC	+250	*ssgG*
ssgR_RTL	CGTGTACTACGCCACGTACC	+269 [b]	*ssgR*
ssgR_RTR	AGCCGCTTCAGGAGACTGT	+508 [b]	*ssgR*
Primers for DACA and EMSA			
sco0608_pETma	AATAGAATTC ATGTCCGAGAGCACGATGC		
sco0608_pET24ma	ATATAAGCTTTCAGCTGCCGTTCCGCGCGG		
ssgAfor	TGGCCATCTCACTGCCCCTCC		
ssgA_Rev_biotin	CGCAGCTCCACCGGGATGCGG		

ssgB_For	TCGTCCGTCCCCGTACGGTCT		
ssgB_Rev_biotinylated	GCAGGTGCAGCTCGCAGCTGA		
ssgG_For	TCGTCCCGTATCTGCTCGG		
ssgG_Rev_biotin	AGCTCCAGCTCGCGTTCCACG		
ssgR_For	GTGAGAGCCGTGGGACAC		
ssgR_Rev_biotin	CACGGAGCCGATCAGAGT		
ssgR_EMSAF	FAMGCGCAGCGACTGGTGAGAG		
ssgR_EMSAR	GTGCGCCATTCGCGGAGTC		
Primers for EMSA (fluorescent probes)			
SCO4119p1	CTACACACCGTGGGAAAGAA	-305	SCO4119
SCO4119p2	FAM-AGCCGCGGGAACCAACGTCG	-126	SCO4119
ssgRp1	GTGAGAGCCGTGGGACAC	-321	*ssgR*
ssgRp2	FAM-AGCGAATGCGGTGTCAG	-97	*ssgR*
actII-4(s2)p1	TTGGGACGTGTCCATGTAATCACC	+15	*act*II-ORF4
actII-4(s2)p2	FAM-TCGTGCCGCCTGAGGAGCAGCAGC	+115	*act*II-ORF4

* Underlined sequences indicate non-homologues sequence added to create restriction sites (in italics) at the ends of the PCR fragments. Restriction sites: GGATCC, BamHI; GAATTC, EcoRI; AAGCTT, HindIII; TCTAGA, XbaI; CTCGAG, XhoI. Positions are relative to the translational start of the indicated gene (for ssgAsg and ssgAsc we refer to the first of the three possible ATG start sites as +1).

[a] based on the re-annotated translational start site for *ssgB*, which lies 66 nt downstream of the start predicted in StrepDB.

[b] based on the translational start site predicted in StrepDB. The true start of *ssgR* is most likely further upstream (see the manuscript).

Table S3. Identification of peptides by DACA assay.

Protein (SCO Reference) *a*	ID	peptide (aa sequence)	MH+ *b*	z	Time point	Xcorrelation score (peptide ID) *c*			
						ssgA	*ssgB*	*ssgG*	*ssgR*
SlbR	1	R.SAAEVTTALSEAHSGR.T	1586,8	2	48h	20.25 (1,3)	15.18* (1,3)		
(SCO0608)	2	K.VTIPGFLTFER.T	1279,7	2	72h				
	3	R.ATAQPTLVELVR.E	1297,7	2					
AdpA	4	R.LLETSDYSVDEVAGR.C	1653,8	2	48h	10.17 (4)	20.17 (4,6)		
(SCO2792)	5	R.LLVCAGEDGPLR.T	1299,7	2	72h	30.2 (4,5,6)	120.20 (4,5)	20.17* (4,5,6)	
	6	R.TTGGLELTAPQGLEAISR.A	1814,0	2					
HupA	7	R.SELVAALADR.A	1044,6	2	48h	15.21* (7,9,10)	15.23* (7,9)	10.21* (9)	50.25 (7,8,9,10)
(SCO2950)	8	K.VTIPGFLTFER.T	1279,7	2	72h	20.21 (7,9)	15.22* (7,9)	10.22* (7,9)	40.25 (7,8,9,10)
	9	R.NPQTGEPIQIPAGYSVK.V	1798,9	2					
	10	R.TARNPQTGEPIQIPAGYSVK.V	2127,1	3					
FruR	11	R.MAAAIPPDASLTVVTHSLPIAAR.L	2302,3	3	48h	10.22 (11)	20.26 (11,14)	10.15 (11)	20.22 (11,12)
(SCO3198)	12	R.LADHPGIQLHIVGGR.V	1582,9	3	72h	20.18 (11/13)		10.19 (11)	
	13	R.ADVAVVAANGFSVEHGLTTPDLAEAAVKR.A	2908,5	3					
	14	R.AALAELPAEGTLILDAGSTVAR.M	2139,2	2					
Lsr2 domain protein	15	K.TYEIDLTTANADK.L	1454,7	2	48h				
(SCO3375)	16	K.TYEIDLTTANADKLR.G	1723,9	2	72h		20.20 (15,16)		
sugar diacid regulator	17	R.FGLVLSHAHAVAVAQGPEAYDDTAPVTR.V	2892,5	3	48h				
(SCO3606)	18	R.AADMLVYPVLAR.D	1318,7	2					
	19	RVAVGRPQSGAGGVVHSYEEALTTLELADRL	2982,5	3	72h	10.19(19)	10.16 (17)		10.15(18)
XRE-family protein	20	R.LIDDLGLNQAR.L	1227,7	2	48h				
(SCO3859)	21	R.VQLLQDLAGQVADGSVSAAEATER.M	2428,2	3	72h	20.17 (20,21)	30.26 (20,21)		
	22	R.SVAAAGDIIDAADSLSSSHPELAEFLR.V	2742,4	3					
AtrA	23	R.TALGQEDEPWSALSR.F	1659,7	2	48h				30.22 (23,24,25)
(SCO4118)	24	R.EVFGELGYGAPMEDVAR.R	1839,9	2	72h				40.22 (23,24,25,26)
	25	R.LLDILLEGLR.S	1154,7	2					
	26	R.VGVSDDAGTDGGVGGPGAAAAGDPR.V	2126,0	3					
LexA, SOS regulator	27	R.GSDQAASVQPTDTAGKPAASYVPLVGR.I	2643,3	3	48h		20.24 (27,28)		
(SCO5803)	28	R.IAAGGPILAEESVEDVFPLPR.Q	2180,2	2	72h		30.22 (27,28,29)		
	29	R.RQPVAENGDIVAAMLDGEATVKR.F	2440,3	3					

Footnotes

a Refers to the StrepDB database reference number

b MH+: Calculated m/z of the peptide with z = 1

c XCorrelation score: cross-correlation peptide score by SEQUEST database search. Peptide numbers between brackets (Refer to column peptide ID). Default threshold of 10 was used in identifying the peptides.

* averaged Xcorr for peptides found in both of two independent experiments
